# Primary osteomyelitis caused by an NDM-1-producing *K. pneumoniae* strain of the highly virulent sequence type 23

**DOI:** 10.1038/emi.2017.43

**Published:** 2017-06-21

**Authors:** Fabrice Compain, Alexis Vandenberghe, Marie Gominet, Nathalie Genel, David Lebeaux, Astrid Ramahefasolo, Isabelle Podglajen, Dominique Decré

**Affiliations:** 1 Microbiology, AP-HP, Hôpital Européen Georges Pompidou, Paris 75015, France; 2 Université Paris Descartes, Sorbonne Paris Cité, Paris 75006, France; 3 Institut National de la Santé et de la Recherche Médicale, Paris 75013, France; 4 UPMC Univ Paris 06 INSERM, CIMI-Paris, UMR 1135, ERL, CNRS 8544, Paris 75013, France; 5 Microbiology, AP-HP, Hôpital Saint Antoine, Paris 75011, France

**Dear Editor,**

Hypervirulent variant of *Klebsiella pneumoniae* (hvKP) is characterized by its hypermucoviscosity and its capacity to cause invasive community-acquired infections in previously healthy young individuals.^1^ The most common clinical presentation is a pyogenic liver abscess with metastatic spread, while various other invasive infections have been reported.^1, 2^ Strains of hvKP belong predominantly to the capsular serotypes K1 and K2^2^ and their genetic background seems to differ from that of multidrug-resistant (MDR) isolates.^3^ The most commonly encountered sequence types (STs) among hvKP include ST23 (associated with serotype K1), ST25, ST65, ST86, ST375 and the recently emerging ST380.^4, 5^

Most hvKP isolated to date are highly sensitive to nearly all antibiotics except ampicillin. However, MDR isolates belonging to virulent clones have been described recently and extended-spectrum β-lactamase (ESBL) producers were reported among hvKP.^6^ Strains belonging to highly virulent clone ST23 and producing the KPC-2 carbapenemase are rare but have been observed on at least three continents.^7, 8, 9^

We describe here the first NDM-1-producing *K. pneumoniae* isolate of capsular serotype K1 belonging to the highly virulent ST23 clone. It caused primary osteomyelitis in a young diabetic patient from Saudi Arabia. The rise of multidrug resistance in a highly virulent strain is a cause of great concern.

A 30-year-old man with unremarkable medical history except type I diabetes mellitus was transferred to our hospital for maxillofacial surgery subsequent to a road accident 2 months earlier and emergency facial surgery in Saudi Arabia. Surgery at our institution consisted in the ablation of all osteosynthesis material as well as osteotomy of the zygomatic arch, maxilla and mandible. Abundant hvKP resistant to all carbapenems was found in all surgical samples and osteomyelitis was diagnosed. Whole-body CT scanning revealed no other foci of infection. The patient was treated with parenteral meropenem, amikacin and fosfomycin for 15 days, followed by ciprofloxacin and sulfamethoxazole/trimethoprim for a total of three months and was discharged in good condition.

Four unique carbapenem-resistant *K. pneumoniae* isolates were obtained from the patient. One isolate was the sole organism cultured from all perioperative samples, while three were found only in systematic rectal swab screening sample obtained at admission (Table 1). The four isolates were high-level resistant to all β-lactams including carbapenems. The isolate responsible for osteomyelitis (KP43-45) remained susceptible to aminoglycosides, ciprofloxacin, sulfamethoxazole/trimethoprim, tigecycline and fosfomycin, while the other isolates showed various associated resistance profiles (Table 1).

PCR and sequencing revealed the presence in all isolates of the carbapenemase *bla*_NDM-1_ gene, associated with the carbapenemase *bla*_OXA-48_ gene in three of them, that is, KP43-53, KP43-54 and KP43-55 (Table 1). ESBL production was detected in all four isolates. Plasmid analysis was performed using the relaxase gene typing method as previously described.^10^ KP43-45 (harboring only *bla*_NDM-1_) contained a plasmid of the FIIk incompatibility (Inc) group, while plasmids belonging to IncFIIk and IncL/M were detected in isolates carrying both *bla*_NDM-1_ and *bla*_OXA-48_.

Multilocus sequence typing (http://bigsdb.web.pasteur.fr/) revealed two distinct allelic profiles, one for KP43-45, KP43-53 and KP43-54 belonging to ST23 and one for KP43-55 belonging to ST437. This was in concordance with molecular fingerprints obtained using random amplified polymorphic DNA and repetitive element palindromic sequence-based PCR (data not shown).

The three hypermucoviscous isolates of capsular serotype K1 belonging to the ST23 clone were string-test positive^1^ and carried the *rmpA* (regulator of mucoid phenotype A), *entB* (siderophore, enterobactin), *ybtS* (siderophore, yersiniabactin), *kfu* (siderophore), *iutA* (siderophore, aerobactin), *mrkD* (type 3 fimbrial adhesin) and *allS* (allantoin metabolism) virulence genes (Table 1). KP43-55 (ST437) was string-test negative, non-K1/K2 and tested positive only for the *entB*, *ybtS* and *mrkD* virulence genes.

Hypervirulent variant *K. pneumoniae* is a globally emerging pathogen that was first described in Taiwan and has now spread to other regions in Southeast Asia, Europe and the Americas.^2, 5^ Invasive syndromes due to hvKP have been reported to occur in the Middle-East. Our patient was repatriated from Saudi Arabia, a country where a case of liver abscess caused by a serotype K1 *K. pneumoniae* isolate was recently described.^11^ The novel *bla*_NDM-1_ -producing isolate described here may well have been acquired in that country.

*K. pneumoniae* ST23 strains belong to a virulent clone that is highly prevalent in *K. pneumoniae* liver abscesses.^2, 4^ Most ST23 strains are hypermucoviscous, which seems to correlate with the presence of the *magA* and *rmpA* genes.^1^ The ST23 strains described in our study also carried seven virulence genes often found in this clone.^3, 4^ A non-hvKP isolate of ST437 was found in a rectal swab sample. ST437 is part of clonal complex 11, a high-risk MDR complex.^12^ A horizontal transfer of resistance genes from the ST437 isolate to the ST23 isolates is highly probable; however, our mating experiments to substantiate this were not successful.

*K. pneumoniae* ST23 isolates have been found to be susceptible to most drugs including third-generation cephalosporins (3GC), fluoroquinolones and aminoglycosides. Although some studies have described ESBL-producing isolates resistant to 3GC or carbapenems,^6^ reports of carbapenemase-producing ST23 isolates remain rare. KPC-2-producing ST23 isolates were described in China and Poland in 2010 and 2011, respectively, but without any clinical or microbiological supplementary information.^8, 9^ A hypermucoviscous KPC-2-producing *K. pneumoniae* belonging to ST23 was recovered in a respiratory sample of a patient from Argentina.^7^ Although carbapenemase-producing Gram-negative bacteria have largely spread in the Arabian Peninsula, no carbapenemase-producing *K. pneumoniae* belonging to ST23 have been described before in that region.^13^

In this study, we describe the first hvKP isolate belonging to ST23 and producing the NDM-1 carbapenemase. This isolate had all the hvKP characteristics described by Shon *et al.*,^1^ as it was responsible for an invasive infection in a previously healthy young patient and displayed particular microbiological features such as hypermucoviscosity and the presence of virulence factors. The only underlying disease that could be identified in our patient was type I diabetes mellitus, which has been suggested in some studies to be a potent risk factor for hvKP infection.^1, 2^ An interesting feature was the intestinal-tract carriage of three unique hypermucoviscous, carbapenem-resistant *K. pneumoniae* isolates, including that responsible for osteomyelitis. Asymptomatic carriage of hvKP isolates has been suggested to be a major risk factor for infection in Asian patients.^14^ Therefore colonization with hvKP ST23 isolates harboring both NDM-1 and OXA-48 carbapenemases with associated resistance to aminoglycosides and ciprofloxacin in our patient should cause concern. Moreover, plasmid typing identified IncFIIk and IncL/M replicons, which are part of conjugative plasmids involved in horizontal transfer of carbapenemase genes.^15^ IncL/M plasmids have been previously associated with the dissemination of the *bla*_OXA-48_ gene.^15^

Acquisition of carbapenemase genes by the hvKP ST23 strains is worrisome and should be closely monitored. This can be done easily with the string test, MLST and identification of virulence factors. The acquisition of *bla*_NDM-1_ might be one step leading to treatment failure in patients infected with hypervirulent *K. pneumoniae*.

## Figures and Tables

**Table 1:** Resistance profile, virulence properties and genetic background of the four *K. pneumoniae* isolates analyzed

**Variable**	**KP 43-45**	**KP 43-53**	**KP 43-54**	**KP 43-55**
Isolate source	Frontal sinus mucocele and sequestra; rectal swab	Rectal swab	Rectal swab	Rectal swab
				
*Antibiotic and disk diffusion zone diameter (mm)*				
AMP	6 (R)	6 (R)	6 (R)	6 (R)
AMC	6 (R)	6 (R)	6 (R)	9 (R)
TZP	6 (R)	6 (R)	6 (R)	11 (R)
CTX	6 (R)	6 (R)	6 (R)	6 (R)
CAZ	6 (R)	6 (R)	6 (R)	6 (R)
ATM	6 (R)	14 (R)	6 (R)	10 (R)
FEP	6 (R)	12 (R)	6 (R)	11 (R)
IPM	16 (I)^a^	17 (I)	18 (I)	16 (I)
MEM	15 (R)^a^	15 (R)	16 (I)	15 (R)
AN	22 (S)	9 (R)	10 (R)	9 (R)
GE	24 (S)	27 (S)	30 (S)	13 (R)
CIP	22 (S)^a^	21 (I)	21 (I)	6 (R)
SXT	20 (S)	23 (S)	27 (S)	6 (R)
FOS	20 (S)	22 (S)	21 (S)	24 (S)
Carbapenemase content	NDM-1	NDM-1, OXA-48	NDM-1, OXA-48	NDM-1, OXA-48
Plasmid Inc type	FIIk	FIIk, L/M	FIIk, L/M	FIIk, L/M
MLST	ST23	ST23	ST23	ST437
Rep-PCR or RAPD profile	A	A	A	B

*Virulence properties*				
String-test	Positive	Positive	Positive	Negative
Capsular polysaccharide	K1	K1	K1	non-K1/K2
Virulence genes	*magA, rmpA, ybtS, kfu, iutA, allS, entB, mrkD*	*magA, rmpA, ybtS, kfu, iutA, allS, entB, mrkD*	*magA, rmpA, ybtS, kfu, iutA, allS, entB, mrkD*	*ybtS, entB, mrkD*

Abbreviations: ampicillin, AMP; co-amoxiclav, AMC; piperacillin-tazobactam, TZP; cefotaxime, CTX; ceftazidime, CAZ; aztreonam, ATM; cefepime, FEP; imipenem, IPM; meropenem, MEM; amikacin, AN; gentamicin, GE; ciprofloxacin, CIP; sulfamethoxazole/trimethoprim, SXT; fosfomycin, FOS; tigecycline, TGC; susceptible, S; intermediate, I; resistant, R, according to EUCAST breakpoints version 5.0 (www.eucast.org); multilocus sequence typing, MLST.

a For the clinical isolate, MIC of ertapenem was of 32 μg/mL, IPM 8 μg/mL, MEM 12 μg/mL, CIP 0.5 μg/mL and TGC 0.75 μg/mL. Minimal inhibitory concentrations were determined with the E-test (bioMérieux).
